# Effects of Health Belief About COVID-19 on Knowledge Sharing: The Mediating Role of Self-Efficacy

**DOI:** 10.3389/fpsyg.2022.882029

**Published:** 2022-07-27

**Authors:** Tianjiao Wang, Cheng Jiang, Qiran Chen

**Affiliations:** Institute of Economics of Education, Peking University, Beijing, China

**Keywords:** health belief, knowledge, self-efficacy, COVID-19, China

## Abstract

While existing studies have explored factors that affect knowledge sharing among employees from different perspectives, there are still research gaps regarding whether health belief affects knowledge sharing among employees, specifically against the backdrop of the COVID-19 pandemic, and how such effects work. Thus, the purpose of this study is to determine the effect of bank employees’ health beliefs about COVID-19 on knowledge sharing mediated by their self-efficacy. From the perspective of social cognitive theory and the health belief model, this study investigates whether employees’ perception of susceptibility and severity of COVID-19 affects formal as well as informal knowledge sharing through knowledge sharing self-efficacy. A sample of 407 bank employees (200 women and 207 men) in China was used for the study. The formulated hypotheses were tested using structural equation modeling and bootstrapping. The results showed that employees’ perceived susceptibility to COVID-19 significantly undermines formal and informal knowledge sharing self-efficacy. However, there was no significant difference in the extent of its indirect effects on formal and informal knowledge sharing. Further, employees’ perceived severity of COVID-19 had no effect on knowledge sharing self-efficacy and on formal and informal knowledge sharing, which could have resulted from the COVID-19 outbreak in China.

## Introduction

Coronavirus disease 2019 (COVID-19) was first declared a pandemic on March 11, 2020, and has caused widespread disruption in all areas of people’s social life since its emergence (World Health Organization, 2020). As of January 2022, more than 370 million confirmed cases and over 5.6 million COVID-19-related deaths have been reported all over the world (World Health Organization, 2021). Since the emergence of the virus in Wuhan in September 2019 (Huang et al., 2020), more than 130,000 people have been diagnosed with the disease in China, as of January 2022 (World Health Organization, 2021). Although the number of COVID-19 cases in China accounts for only a small proportion of the country’s overall population, the virus poses a huge burden on the country (Li et al., 2020; Zhong et al., 2020). As far as business organizations are concerned, the massive impact of the pandemic has also affected Chinese companies. Many companies suffered a huge existential crisis during the pandemic (Wang et al., 2020; Ng et al., 2022). Meanwhile, people have been forced to adopt a series of measures to avoid infection, such as wearing masks, keeping social distance, or even working from home, which have adversely affected their work and life (Li et al., 2021a; Aldianto et al., 2021).

The organizational crises and individual psychological stress have posed additional challenges for knowledge sharing among employees (Lee et al., 2021) during COVID-19 (Xu et al., 2021). Existing studies have shown that knowledge sharing can bring innovative ability or sustainable benefits to companies (Ruuska and Vartiainen, 2005; Cerne et al., 2014; Škerlavaj et al., 2018). Particularly, the role of knowledge sharing in organizational development is crucial for knowledge-intensive organizations (Henttonen et al., 2016). As a typical knowledge-intensive organization, knowledge sharing is even more important for banks. In the context of COVID-19, a new crisis has arisen in the form of knowledge sharing within companies. Organizational crises can also lead to persistent negative emotions among employees within companies (Konig et al., 2020) and further inhibit knowledge sharing (Nguyen et al., 2022).

Given the crucial role of knowledge sharing in the sustainable development of companies (Kogut and Zander, 1996; Argote and Ingram, 2000; Wang and Noe, 2010), there is a need to analyze how the pandemic affects knowledge sharing among employees. Previous studies have investigated some factors affecting knowledge sharing and how their effects work at the individual level (Bock et al., 2005; Wasko and Faraj, 2005; Chiu et al., 2006; Israilidis et al., 2015). These factors include anticipated reciprocal relationships (Bock et al., 2005; Wasko and Faraj, 2005; Chiu et al., 2006), trust (Nahapiet and Ghoshal, 1998; Wen and Wang, 2021), image (Wasko and Faraj, 2005), identification (Kankanhalli et al., 2005), organizational rewards (Bock et al., 2005), perceived loss of knowledge (Davenport and Prusak, 1998), and knowledge self-efficacy (Jarvenpaa and Staples, 2000; Bock et al., 2005; Wen and Wang, 2021) are deemed to have a significant effect on knowledge sharing among employees. As employees’ health belief and knowledge sharing are likely to have been highly affected during COVID-19, we conducted a literature search on the areas. Nevertheless, studies on the relationship between health belief and knowledge sharing among employees during the COVID-19 pandemic are few. Although a significant correlation between health belief about COVID-19 and self-efficacy among individuals has been confirmed in a recent study (Talsma et al., 2021), managers might fail to correctly explain the reasons behind a decline in employees’ enthusiasm for knowledge sharing **during the pandemic** due to unavailable research in this area. From the perspective of the health belief model, perceived susceptibility and perceived severity of COVID-19 might cause employees to develop negative emotions regarding knowledge sharing, which might be mediated by self-efficacy. According to previous studies, self-efficacy was also used as a mediating variable in some models (Honicke and Broadbent, 2016; Parhamnia et al., 2021). Furthermore, employees with high levels of perceived susceptibility and perceived severity also demonstrate more negative attitudes when communicating and collaborating with their colleagues, which can result in poor knowledge sharing self-efficacy. Meanwhile, the positive relationship between self-efficacy and employee behavior is an important concern in social cognitive theory. Consequently, the following research question guides this study—does employees’ health beliefs about COVID-19 influence their knowledge sharing self-efficacy?

Based on the above discussion, this study proposes the effects of health beliefs about COVID-19 on knowledge sharing among employees and the mechanism behind such effects from the perspective of bank employees’ self-efficacy. Specifically, this study considered perceived susceptibility and perceived severity, two key factors in the health belief model (Janz and Becker, 1984; Yuen et al., 2020), as independent variables and analyzed them using structural equation modeling. The aim was to determine whether these two factors affect formal and informal knowledge sharing among bank employees under the mediating role of self-efficacy. The contributions of this study are threefold. First, this study combines the perspectives of the health belief model and social cognitive theory to analyze the effects of health belief on knowledge sharing in the context of the COVID-19 pandemic, thereby broadening research perspectives on the factors affecting knowledge sharing. Second, this study enriches the differentiated understanding of the factors affecting knowledge sharing by taking into consideration different types of knowledge sharing and analyzing the mechanisms behind the formation of formal knowledge sharing and informal knowledge sharing. Finally, this study is of high academic value in research on employees in the banking sector; a large commercial bank in China, which is a knowledge-intensive firm with a large number of employees, is chosen for this study.

This paper is structured as follows. The next section features a review of existing literature and proposes the corresponding research hypotheses. The third section provides an overview of the research methods used in this study, including the process of data collection and questionnaire development. The fourth section reports the results of model analysis. The fifth section discusses the results of this study, and presents the theoretical and temporal implications of this study as well as its limitations.

## Literature Review and Hypotheses

### Knowledge Sharing

In knowledge-intensive organizations, knowledge sharing is important (Henttonen et al., 2016), which is reflected in the processes and results of the organization’s operations. In processes, knowledge sharing can advance organizational knowledge creation (Nonaka, 1994; Kogut and Zander, 1996) and innovation (Cohen and Levinthal, 1990) by increasing the efficiency of collective knowledge utilization. In results, effective sharing of relevant specialist knowledge can help the organization gain a competitive advantage and sustained performance (Kogut and Zander, 1996; Argote and Ingram, 2000; Wang and Noe, 2010). It also influences employees’ job performance and job satisfaction, especially for engineers (Kianto et al., 2016). Even if it introduces organizational challenges (Mabey and Zhao, 2017; Donnelly, 2019), it also plays a key role in sustaining knowledge-based companies’ success and competitiveness (Cegarra-Navarro et al., 2016; Kianto et al., 2017). While the importance of knowledge sharing is self-evident, knowledge sharing strategies are often overlooked in general business strategies, as it is challenging to assess its value effect and action mechanism (Yao et al., 2020). Although knowledge sharing is a common topic in the field of knowledge management research, there are still gaps in the discussion on its influencing factors and formation mechanism.

Knowledge management refers to identifying and leveraging the collective knowledge in an organization to help the organization compete (Von Krogh, 1998). According to Szulanski (1996) and Bhatt (2001), knowledge sharing generally refers to moving knowledge between different organizational actors, both within and between departments and hierarchical levels. In organizations, the key objective of knowledge sharing between employees is the systematic translation of existing knowledge into organizational resources (Dawson, 2001). Within a larger intellectual framework, knowledge sharing is a crucial ingredient of knowledge management at both the organizational and individual levels (Riege, 2005; Oyemomi et al., 2019; Singh et al., 2019). Typically, knowledge management is seen to consist of knowledge processes (such as knowledge creation, sharing, acquisition, transfer, and application) together with infrastructures, capabilities, and management activities that support and enhance the knowledge processes (Gold et al., 2001; Lee and Choi, 2003). Specifically, knowledge sharing is focused on maximizing the utility of collective knowledge. The study of its influencing factors and pathway mechanisms will provide a new theoretical perspective on knowledge management within organizations; it could even help to improve the knowledge management level of cross-organizational cooperation (Wen and Wang, 2021).

With the expansion of knowledge management theory, knowledge sharing, as one of the important micro-concepts, has been enriched in different aspects. From the participant’s aspect, there is individual knowledge sharing, group knowledge sharing, and organizational knowledge sharing (Nonaka and Takeuchi, 1995). From the ontological aspect, there is a group (team) knowledge sharing, organizational knowledge sharing, and inter-organizational knowledge sharing; from the epistemological aspect, there is explicit knowledge sharing and tacit knowledge sharing (Liao et al., 2007; Lin, 2007a; Yesil et al., 2013). From the aspect of the knowledge source, there is internal knowledge sharing and external knowledge sharing (Carmeli et al., 2013).

Furthermore, referring to the distinction between formal and informal knowledge management proposed by Hutchinson and Quintas (2008), knowledge sharing could also be divided into formal knowledge sharing and informal knowledge sharing (Witherspoon et al., 2013; Kumari and Takahashi, 2014; Park and Kim, 2018; Lee et al., 2020) from the perspective of the knowledge sharing process. Formal knowledge sharing refers to the storage and exchange of knowledge that takes place publicly within the formal rules and structures of organizations whereas informal knowledge sharing is the exchange of knowledge about one’s daily work among colleagues and in highly private settings (Lee et al., 2020).

Although formal knowledge sharing and informal knowledge sharing are both under the theoretical framework of knowledge sharing, there are significant differences in their mechanisms of influence. According to the research results (Mitchell et al., 2014), it verifies effectiveness by organization size. For example, formal knowledge sharing plays a key role in the performance of small- and medium-sized enterprises. It connects their knowledge with and develops the globally advanced knowledge system by transferring innovative knowledge and technology and sharing specific local experiences. Compared with formal knowledge sharing, informal knowledge sharing is a more flexible knowledge sharing practice (Biancani et al., 2014). Informal knowledge sharing accounts for most KS activities even in a highly institutionalized KS organization (Krogh et al., 2000), and it can occur without specific intentions (Swap et al., 2001). On the other hand, another study shows that both formal knowledge sharing and informal knowledge sharing positively contribute to the task performance of manufacturing companies (Wen and Wang, 2021).

Based on the above discussion, it could be concluded that although some literature makes a distinction between formal knowledge sharing and informal knowledge sharing, the discussion of the differences is not sufficient so far. Besides, available studies have focused on the differences in the effects of formal knowledge sharing and informal knowledge sharing, without noting the differences in the formation mechanisms of formal knowledge sharing and informal knowledge sharing.

### Health Beliefs About COVID-19 and Self-Efficacy

Against the backdrop of the COVID-19 pandemic, this study aimed to investigate the effects of health beliefs on knowledge sharing self-efficacy among employees. In the health belief model, perceived susceptibility and perceived severity are the two key factors of greatest concern (Janz and Becker, 1984; Yuen et al., 2020). While perceived susceptibility refers to an individual’s vulnerability under a particular health risk, perceived severity refers to an individual’s perception of the danger of a particular health risk (Carpenter, 2010; Timpka et al., 2014). Based on the health belief model, the health belief about COVID-19 refers to the individual’s vulnerability to COVID-19 and the individual’s perception of the risk of COVID-19.

In existing studies, perceived susceptibility and perceived severity have been found to have a significant effect on an individual’s attitude and behavior (Wang et al., 2021). Moreover, health belief about COVID-19 was also found to have a strong association with self-efficacy (Talsma et al., 2021).

As a key element in social cognitive theory, self-efficacy is an individual’s “can do” belief about a future performance outcome (Bandura et al., 1997). In organizational management-related research, knowledge sharing self-efficacy is defined as an employee’s confidence in his/her ability to provide valuable knowledge to other employees in the organization (Spreitzer, 1995; Nguyen and Malik, 2020). As regards the effects of health belief about COVID-19 on self-efficacy, change in the emotional state serves as a critical path (Bandura et al., 1997). Existing studies argued that individuals use feelings such as stress, fatigue, anxiety, and uncertainty as cues to judge self-efficacy (Usher and Pajares, 2006). For example, employees lose confidence in the value of knowledge sharing and lower their expectations of the effectiveness of knowledge sharing when they feel stressed about knowledge sharing activities. Conversely, employees become more confident in their abilities when they are in a calm emotional state (Tan et al., 2021). Therefore, employees are more likely to develop negative emotions such as stress and anxiety during knowledge sharing when they believe that their environment is threatened by COVID-19 (Petzold et al., 2020; Losada-Baltar et al., 2021), where such emotions are deemed to have a negative association with self-efficacy (Young et al., 2008). Without a doubt, some studies also suggested that self-efficacy can counter the effects of external environments to a certain extent (Bong, 2002; Foster et al., 2016). However, a growing number of studies have shown significantly higher levels of negative emotions among individuals during the COVID-19 pandemic (Han et al., 2021; Rana and Islam, 2021), which will challenge the ability to regulate self-efficacy.

In the wake of multiple shutdowns and continuous social distancing during the COVID-19 pandemic, employees’ vulnerability to COVID-19 infection and concerns about the serious consequences of COVID-19 infection can also reduce the frequency and quality of communication among employees (Rfg et al., 2021). Considering that the key goal of knowledge sharing is to transfer knowledge quickly and effectively between individuals (Wen and Wang, 2021), reduced communication efficiency can ultimately diminish employees’ confidence in the value of knowledge sharing, which in turn is manifested as a reduction in self-efficacy. To the best of our knowledge, no study has so far measured health belief about COVID-19 among employees in Chinese commercial banks and investigated its effects on knowledge sharing self-efficacy. Therefore, based on social cognitive theory, the following hypotheses are proposed in this study:

*H1a*: Perceived susceptibility negatively affects knowledge sharing self-efficacy among employees.

*H1b*: Perceived severity negatively affects knowledge sharing self-efficacy among employees.

### Self-Efficacy and Knowledge Sharing

Social cognitive theory suggests that an individual’s self-efficacy affects his/her behavior in multiple ways (Bandura et al., 1997). As regards knowledge sharing, which is the focus of this study, existing research also showed that self-efficacy can significantly affect employees’ knowledge sharing intention and behavior (Chen et al., 2012; Nguyen and Malik, 2020; Wen and Wang, 2021). A higher level of self-efficacy tends to correspond to a higher level of self-motivation (Hsu et al., 2007) which facilitates increased enthusiasm for knowledge sharing among individuals (Bock and Kim, 2002; Lin, 2007b). On top of that, some scholars argued that employees actively share knowledge because they believe that their knowledge can help their colleagues solve problems effectively (Bysted, 2013); these kinds of employees usually have high confidence in their ability to complete tasks (Shao et al., 2015).

In empirical research, Teh et al. (2010) who developed a framework based on the theory of planned behavior, found that media self-efficacy has a significant positive effect on knowledge sharing through knowledge sharing intention. Among studies in the Chinese context, Yang and Xu (2021) also discovered that employees’ self-efficacy promotes knowledge sharing behavior through feelings of job security and knowledge sharing intention. In a recent survey conducted among manufacturing employees, Wen and Wang (2021) found that self-efficacy does not only positively affects informal knowledge sharing and formal knowledge sharing but also promotes employees’ task performance. Despite extensive investigations into the relationship between self-efficacy and knowledge sharing in existing studies, the relationships between self-efficacy and different forms of knowledge sharing have not been fully explored and require analysis in a broader social and industry context. Therefore, based on the theory of planned behavior, the following hypotheses are proposed in this study:

*H2a*: knowledge sharing self-efficacy negatively affects informal knowledge sharing among employees.

*H2b*: knowledge sharing self-efficacy negatively affects formal knowledge sharing among employees.

## Research Method

### Sample and Data

The data used for this study were collected using a questionnaire designed for a survey participated by 32 branches of a large commercial bank in China. The questionnaire was first distributed to 15 bank employees for preliminary testing after it was developed. Then, a few questions were adjusted based on feedback collected from these employees to eliminate ambiguities. According to the feedback collected during preliminary testing, the questionnaire was found to have high overall reliability and acceptability. Next, with the support of the bank’s human resources department, an online questionnaire was sent to the corporate e-mail addresses of 700 bank employees between December 28, 2021, to January 2, 2022. During the questionnaire distribution period, a total of 564 employees voluntarily filled out and submitted the questionnaire, indicating an 80.6% response rate in the process. To ensure the quality of the survey data, questionnaires that were filled out over an overly short period (less than 200 s) and those which failed the attention checker process were removed from the sample. In the end, a total of 407 valid questionnaires were obtained, representing a 72.2% valid response rate.

Table 1 summarizes the sample profiles. According to the statistical results, 49.1% of the respondents in the sample are female and 50.9% are male, with the majority of the respondents aged between 26 and 55 years old, accounting for 92.8% of the respondents in the sample. In terms of education degree, all the respondents have received higher education, with 94.3% of them holding a Bachelor’s or a Master’s degree, which is higher than the percentage of higher education degree holders among Chinese nationals (15.46%). This is because large commercial banks are more attractive to highly educated job seekers and these banks, which are also knowledge-intensive companies, also prefer to recruit highly educated talents (King et al., 2016). As for job function, the sample comprised employees at all levels in the bank, with general staff constituting the largest proportion at 63.9%.

**Table 1:** Sample profile.

		Frequency	Proportion (%)
Gender	Female	200	49.1
	Male	207	50.9
Age	<26	23	5.7
	26–35	180	44.2
	36–45	123	30.2
	46–55	75	18.4
	>55	6	1.5
Degree	Junior college	23	5.7
	Bachelor	305	74.9
	Master	79	19.4
	Other	0	0
Job function	Top managers	6	1.5
	Middle managers	69	17.0
	First-line managers	72	17.7
	General staff	260	63.9

To test whether the questionnaire results in common method bias, we performed factor analysis on all items using Harman’s single-factor test (Podsakoff and Organ’s, 1986; Sharma et al., 2009). According to the results of factor analysis performed using SPSS Statistics, version 22, the first factor was able to explain approximately 34.42% of the total variance, which does not exceed 50%; in other words, this finding proves that common method bias, which threatens the validity of the survey, is not present in this study (Harman, 1976; Sharma et al., 2009).

### Measurements

The introduction section of the questionnaire generally explained the purpose of this study to the respondents and guaranteed that the questionnaire does not cover any individual evaluation or employee performance appraisal items and will be used for academic research only. Furthermore, it explained that the survey data will be kept confidential. All the questionnaire items were measured using a seven-point Likert scale, where 1 = “completely disagree” and 7 = “completely agree.” The original version of the questionnaire was written in English while the Chinese version of the questionnaire was developed by two linguists after translating the questionnaire items into Chinese. Then, the Chinese version of the questionnaire was re-translated into English by native English speakers to ensure consistency in the meaning of the scale across different language versions. The final constructs obtained during the preliminary survey were re-translated into English as well and their sources of reference are listed in Table 2.

**Table 2:** Construct reliabilities and AVE.

Constructs	Items	Standardized factor loadings	Cronbach α	AVE	CR
Formal knowledge sharing	Formal knowledge sharing 1	0.919	0.867	0.832	0.952
	Formal knowledge sharing 2	0.905			
	Formal knowledge sharing 3	0.939			
	Formal knowledge sharing 4	0.884			
Informal knowledge sharing	Informal knowledge sharing 1	0.868	0.859	0.838	0.954
	Informal knowledge sharing 2	0.943			
	Informal knowledge sharing 3	0.927			
	Informal knowledge sharing 4	0.923			
Self-efficacy	Self-efficacy1	0.907	0.866	0.88	0.967
	Self-efficacy2	0.973			
	Self-efficacy3	0.962			
	Self-efficacy4	0.907			
Perceived susceptibility	Perceived susceptibility1	0.902	0.839	0.761	0.927
	Perceived susceptibility2	0.900			
	Perceived susceptibility3	0.809			
	Perceived susceptibility4	0.874			
Perceived severity	Perceived severity1	0.887	0.761	0.792	0.919
	Perceived severity2	0.877			
	Perceived severity3	0.905			

*N = 407; AVE, average variance extracted; CR, composite reliability*.

In this study, knowledge sharing behaviors among employees were the dependent variables. While knowledge sharing has been discussed extensively in previous studies (Szulanski, 1996; Bhatt, 2001; Wen and Wang, 2021), further research is needed to investigate the varying factors affecting different forms of knowledge sharing (Nguyen and Malik, 2020). Hence, the dependent variables in this study include two types of knowledge sharing, namely informal knowledge sharing and formal knowledge sharing, and the questionnaire items for these variables were developed by Wen and Wang (2021) and Zahra et al. (2007). The sample items include “My colleagues and I have formal knowledge exchange channels (e.g., routine meetings and project reports)” and “My colleagues and I often share information about ‘changes in customer needs’ through formal channels.”

Meanwhile, the independent variables in this study comprise employees’ perceived susceptibility and perceived severity of COVID-19, which are the two core dimensions of greatest concern in the health belief model (Janz and Becker, 1984; Yuen et al., 2020). Given that the questionnaire items for these variables were developed by Wang et al. (2021), the sample items for perceived susceptibility include “I think I am more likely to contract COVID-19 than others” and the sample items for perceived severity include “My career will be at risk if I contract COVID-19.”

Conversely, the moderating variable in this study is employees’ self-efficacy in knowledge sharing; the questionnaire items developed by Bock et al. (2005) are employed to measure employees’ confidence in knowledge sharing. The instructions read as follows, “When sharing knowledge, I believe that my abilities can ……,” and the sample items include “help my colleagues solve their problems at work” and “help my department improve its workflow.”

## Results

In recent years, a host of quantitative studies have replaced the traditional regression method of estimation with structural equation modeling (Razzaq et al., 2019) as this method can describe the linear relationship between latent variables (Wen and Wang, 2021). The hypotheses proposed in this study were tested using Amos 23.0 through three main steps. First, a preliminary model analysis was carried out on the reliability, convergent validity, and discriminant validity of the model in this study. Next, the hypotheses proposed in the theoretical model were tested using the maximum likelihood method. Lastly, based on structural equation modeling, the bootstrap method was employed to test the strength and significance of the mediating effect *via* repeated sampling.

### Preliminary Model Analysis

Precise results are based on a high level of construct reliability and validity. The Cronbach’s *α* values of all constructs reflected the reliability of the questionnaire. The construct reliabilities are considered good if the value of *α* is larger than 0.70 (Hair et al., 2010). Table 2 shows that the Cronbach’s *α* value ranges from 0.761 to 0.867, which confirms the high construct reliability of our study.

As for the construct validity, it represents the extent to which the items of the questionnaire can measure the theoretical structure and characteristics of key variables. It could be evaluated by convergent validity and discriminant validity, which represent the stability of results and diversity of dimensions (Fornell and Larcker, 1981; Chin et al., 2003).

Convergent validity is commonly reflected by the standardized factor loading, composite reliability (CR), and average variance extracted (AVE; Fornell and Larcker, 1981). Each of these indicators has a corresponding range of values. Specifically, the factor loading should be greater than 0.5 and preferably exceed 0.7, the AVE value should be greater than 0.5, and the CR value should be greater than 0.7 (Hair et al., 2010; Goswami and Agrawal, 2018). Table 2 provides us with relevant evidence. There are five constructs, including formal knowledge sharing, informal knowledge sharing, self-efficacy, perceived susceptibility, and perceived severity. For all five constructs, the standardized factor loadings are greater than 0.80 (>0.7), the AVE is greater than 0.76 (>0.5), and the CR is greater than 0.91(>0.7). So all indicators are acceptable, and construct validity is verified.

Discriminant validity is acceptable if a construct’s square root of the AVE is greater than its correlations with other constructs (Chin et al., 2003). Table 3 provides relevant evidence. The correlation coefficients between the constructs are all less than 0.5, and all coefficients are significant at the 1% level, also less than the square root of the AVE. Therefore, discriminant validity is verified; hence, all latent variables have significant distinctions.

**Table 3:** Discriminant validity results.

	Formal knowledge sharing	Informal knowledge sharing	Self-efficacy	Perceived susceptibility	Perceived severity
Formal knowledge sharing	0.832				
Informal knowledge sharing	0.368^***^	0.838			
Self-efficacy	0.493^***^	0.384^***^	0.880		
Perceived susceptibility	−0.168^**^	−0.035	−0.198^**^	0.761	
Perceived severity	−0.100^*^	−0.040	−0.099^*^	0.465^***^	0.792
Sqr(AVE)	0.912	0.915	0.938	0.872	0.890

*N = 407. The value on the diagonal is AVE*.

*** *p < 0.001;*

** *p < 0.01; and*

* *p < 0.05.*

### Structural Model and Hypothesis Testing

#### Results of Model Fitting

**Figure 1:**
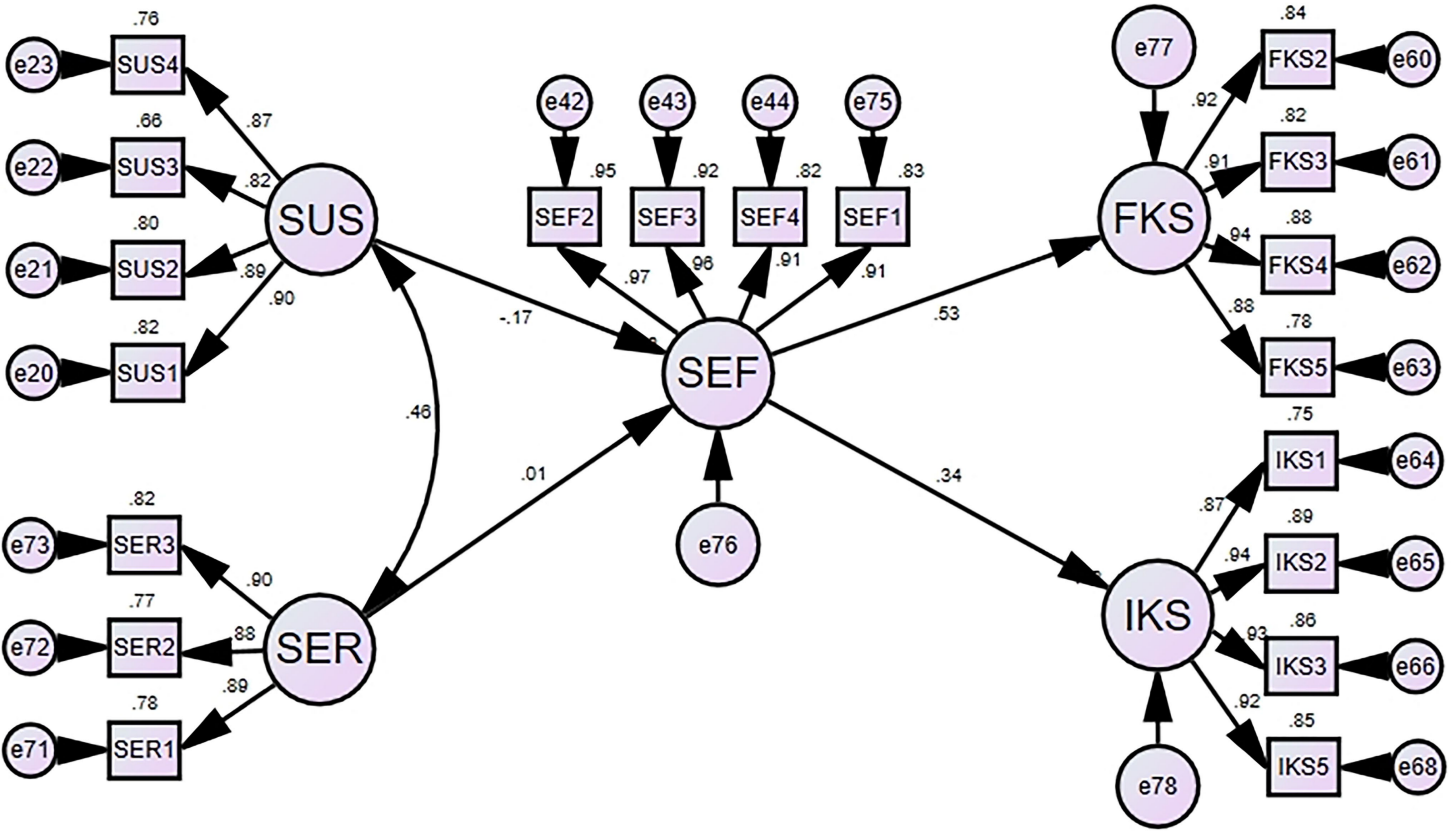
Structural model algorithm.

This study attempted to explore the linear relationship between variables and the path of influence by building structural equation modeling based on the previous hypothesis. There were five constructs and 19 indicator variables in this study. For dependent variables, there were two constructs. Both formal knowledge sharing level and informal knowledge sharing level had four indicators each. For this study, we set the self-efficacy intermediate variables with four indicators. We also set the perceived susceptibility and perceived severity as independent variables, having four and three indicators, respectively. The measurement model is depicted in Figure 1, which presents the result of structural equation modeling and path analysis through AMOS 23.0 software.

Before hypotheses testing, a goodness-of-fit test is necessary. According to confirmatory factor analysis goodness-of-fit statistics of first-order factor model analysis, the model is capable of identifying convergence. The several fit indices considered in this study are related to absolute fit measures, incremental fit indices, and parsimony fit indices, as shown below (Table 4). The results of indices were acceptable. Based on the goodness-of-fit test of the statistical model, the developed hypotheses were tested by the following indicators: hypothesized paths, standardized regression coefficients, and hypothesis testing statistics.

**Table 4:** Goodness-of-fit indices.

Model fit index	Measurement statistics	Recommended range
Absolute fit measures		
GFI	0.923	>0.90
RMSEA	0.054	<0.08
Incremental fit indices		
NFI	0.961	>0.90
CFI	0.975	>0.90
RFI	0.955	>0.90
Parsimony fit indices		
AGFI	0.901	>0.90
PNFI	0.826	>0.50

Sources: Carmines and McIver (1981); Browne and Cudeck (1992); Hu and Bentler (1999).

#### Results of Hypotheses Testing

According to the Table 5, all hypothesized paths are supported except for H1b (*t* = 0.101, *p* > 0.05). The first hypothesized path (H1a) assumes a negative relationship between the level of perceived susceptibility and self-efficacy, which was proved by a significant *t*-value of −2.936 associated with *p* < 0.05. The second hypothesized path (H1b) assumes a positive relationship between the level of perceived severity and self-efficacy. However, it could not be proved by the *t*-value of 0.101 associated with *p* > 0.05. Moreover, hypothesized paths (H2a and H2b) assume positive relationships between both self-efficacy and formal knowledge sharing and self-efficacy and informal knowledge sharing. Both the paths were proved by a significant *t*-value of 11.431 and 6.829 associated with *p* < 0.01. The results of indices are acceptable, which proves that most hypotheses in this study are valid. Perceived susceptibility and self-efficacy have direct or indirect impacts on formal knowledge sharing and informal knowledge sharing. Self-efficacy plays an important mediating role in this influence mechanism.

**Table 5:** Hypotheses testing results.

No.	Hypothesized path	Standardized path coefficient	*t*-Value (C.R.)	Decision
H1a	Perceived Susceptibility→Self-efficacy	−0.174	−2.936^**^	Supported
H1b	Perceived Severity→Self-efficacy	0.006	0.101	Not supported
H2a	Self-efficacy→Formal Knowledge Sharing	0.534	11.431^***^	Supported
H2b	Self-efficacy→Informal Knowledge Sharing	0.341	6.829^***^	Supported

*N = 407.*

*** *p < 0.001;*

** *p < 0.01; and*

* *p < 0.05*.

#### Mediation Analysis

Based on the mediation effect testing, the results in Table 5 validated the direct effects of perceived susceptibility on self-efficacy with a coefficient of −0.174, as well as the direct effects of self-efficacy on formal knowledge sharing and informal knowledge sharing with a coefficient of 0.534 and 0.341, respectively. Thus, self-efficacy mediates perceived susceptibility’s effects on formal knowledge sharing and informal knowledge sharing. It could be concluded that self-efficacy played a significant positive intermediary role between perceived susceptibility and dependent variables (formal knowledge sharing and informal knowledge sharing). The bootstrapping procedure was employed to examine the indirect effects between and across the variables. Based on a sample of 2000 replicates, we calculated confidence intervals at the 95% level. If the confidence interval does not include 0, then the mediate effect can be considered significant. With this widely accepted principle, we can conclude the following result.

For the dependent variable formal knowledge sharing, the results (Table 6) showed that the perceived susceptibility has significant indirect negative effects on formal knowledge sharing with an impact coefficient of −0.09. Meanwhile, the impact of perceived severity on formal knowledge sharing is not significant because the confidence interval includes 0. It is difficult to say that there is any positive or negative effect. For the dependent variable informal knowledge sharing, the results (Table 6) showed that the perceived susceptibility has significant indirect negative effects on informal knowledge sharing with an impact coefficient of −0.058 whereas the effect of perceived severity is too weak to be identified. Hence, the impact of perceived severity on informal knowledge sharing is not significant.

**Table 6:** Mediate effects testing.

Dependent variable	Construct	Indirect effects	Lower bounds	Upper bounds
Formal knowledge sharing	Perceived susceptibility	−0.09	−0.169	−0.016
	Perceived severity	0.005	−0.039	0.043
Informal knowledge sharing	Perceived susceptibility	−0.058	−0.133	−0.013
	Perceived severity	0.003	−0.029	0.034

*N = 407*.

The following conclusions were drawn. First, perceived susceptibility negatively affects both formal knowledge sharing and informal knowledge sharing mediated by self-efficacy. Second, neither the direct effect of perceived severity on self-efficacy is significant nor is the indirect effect of perceived severity on dependent variables (formal knowledge sharing and informal knowledge sharing). The results of the above tests were almost similar to the results of previous literature and the results of our study and were in line with the theoretical framework set out in this study.

## Discussion

The purpose of this study was to analyze the effects of bank employees’ health belief about COVID-19 on knowledge sharing. The study also adopted the health belief model (Janz and Becker, 1984) and social cognitive theory (Bandura, 1985) to investigate the mediating role of self-efficacy in the effects of health belief on knowledge sharing. The proposed hypotheses were tested using structural equation modeling which is an empirical research method widely recognized by the academic field (Thungjaroenkul et al., 2016). A total of 407 employees working in a large Chinese commercial bank participated in the study. The results showed that three hypotheses were supported. Bank employees’ perceived susceptibility to COVID-19 had a significant negative effect on self-efficacy and indirectly undermined formal knowledge sharing and informal knowledge sharing through self-efficacy. Based on the previous researches review, it can be seen that social cognitive theory and self-efficacy have featured prominently in research on the factors affecting knowledge sharing. It also means that choosing this factor for this research contributes to development of social cognitive theory and also guides bank management practices in a health crisis.

The following findings provide several theoretical and practical contributions to existing literature. For theoretical aspects, this study organically combines the health belief model and social cognitive theory to investigate the vital issue of knowledge sharing among employees against the backdrop of the COVID-19 pandemic. It also expands the understanding of the dangers of the COVID-19 pandemic by exploring how employees’ perceptions of the pandemic affect knowledge sharing from the perspectives of perceived susceptibility and perceived severity, even though a vast majority of these employees never actually contracted COVID-19. Although attention has been given to knowledge sharing among employees against the backdrop of the COVID-19 pandemic (Kogut and Zander, 1996; Argote and Ingram, 2000; Wang and Noe, 2010), the effects of health belief factors on knowledge sharing have been largely overlooked. This study discovers that perceived susceptibility, one of the core factors in the health belief model, has a significant negative effect on knowledge sharing self-efficacy and indirectly undermines formal knowledge sharing and informal knowledge sharing among employees. It is noteworthy that the hypothesis stating that perceived severity affects formal knowledge sharing and informal knowledge sharing through knowledge sharing self-efficacy is not supported by the data.

While it is difficult to determine the reasons the hypothesis is not supported. Combining China’s practical experience and strict epidemic prevention measures (Liao and Wang, 2021), one possible explanation is that employees in Chinese companies have a relatively low chance of being exposed to infected individuals’ despite being impacted by the COVID-19 pandemic in numerous other ways. In other words, most employees lack a clear understanding of the consequences of contracting COVID-19 and have difficulty connecting perceived severity to knowledge sharing self-efficacy. Relatively speaking, under the intensive and slender publicity (Li, et al., 2021b) and influence of epidemic prevention policies, which is quite different from the situation in other countries (Teslya et al., 2020), the clearer the employees’ perception of the risk of COVID-19 infection, the more susceptible their psychological state and behavior are to perceived susceptibility.

Furthermore, this study considers different types of knowledge sharing while investigating the effects of health beliefs on formal knowledge sharing and informal knowledge sharing. In recent years, various studies are focusing on the varying antecedents and consequences between different types of knowledge sharing (Yang, 2010; Aubke et al., 2014; Chugh et al., 2021), but have yet to adequately explore differences in their formation from the health belief perspective. Overall, this study finds no significant difference in the effects of health belief on formal knowledge sharing and informal knowledge sharing. There is no statistical significance in the indirect effects of perceived severity on formal knowledge sharing and informal knowledge sharing, whereas the indirect effects of perceived susceptibility on formal knowledge sharing and informal knowledge sharing are significant within the 95% confidence interval. Although the estimated coefficients for the effect of perceived susceptibility on formal knowledge sharing are greater than those for the effect of perceived susceptibility on informal knowledge sharing, the 95% confidence interval for both types of knowledge sharing highly overlaps each other. Thus, the evidence is not sufficient to suggest that there is a significant difference in the extent of the effects of perceived susceptibility on both types of knowledge sharing. Undoubtedly, differences in coefficients still deserve attention. Since formal knowledge sharing is often present in more formal settings, a decline in knowledge sharing self-efficacy caused by perceived susceptibility may put more pressure on employees to engage in formal knowledge sharing in formal settings (Alajmi, 2012; Connelly et al., 2014), thereby leading to a more significant reduction in knowledge sharing self-efficacy compared to informal knowledge sharing. Hence, such differences warrant further investigation in the future.

## Limitations

Despite the strength of this study, there are some obvious limitations in the study. First, countries around the world have developed different anti-epidemic measures in response to the COVID-19 pandemic depending on the extent of the outbreak across countries. This may lead to different levels of risks and impacts of COVID-19 across countries and among different company employees. Therefore, temporal and regional effects should be considered in future studies. Secondly, this study was conducted in the context of extremely stringent disease prevention measures in China, which influenced respondents’ perceptions of health risks, and in turn led to significant differences in the effects of perceived susceptibility and perceived severity on self-efficacy and knowledge sharing. However, the effects of health belief on knowledge sharing may be influenced by other factors, such as cynicism, job insecurity, and role conflict (Nguyen and Malik, 2020). Thus, future studies may extensively consider these factors to enrich the understanding of the relationship between health belief and knowledge sharing.

## Conclusion

Overall, Chinese bank employees’ perceived susceptibility to COVID-19 is found to significantly undermine formal knowledge sharing and informal knowledge sharing through knowledge sharing self-efficacy against the backdrop of the COVID-19 pandemic. Relatively speaking, this study finds no significant effect of perceived severity on knowledge sharing, which may be as a result of the pattern of China’s response to the COVID-19 pandemic and the actual infection situation in the country. These findings add to scholars’ understanding of the relationships of the pandemic with knowledge sharing self-efficacy and knowledge sharing among employees. Furthermore, this study suggests that managers should take into full consideration health belief-related factors when they observe a decline in knowledge sharing among employees. They should also respond proactively from the perspective of psychological intervention and focus on changes in formal knowledge sharing activities to provide full support for formal knowledge sharing among employees in the event of a pandemic.

## Data Availability Statement

The raw data supporting the conclusions of this article will be made available by the authors, without undue reservation.

## Ethics Statement

Ethical review and approval were not required for the study on human participants in accordance with the local legislation and institutional requirements. Written informed consent for participation was not required for this study in accordance with the national legislation and the institutional requirements.

## Author Contributions

TW and CJ: conceptualization, project administration, and resources. TW: formal analysis and methodology. CJ: supervision. TW and QC: writing—original draft and writing—review and editing. All authors have read and agreed to the published version of the manuscript.

## Conflict of Interest

The authors declare that the research was conducted in the absence of any commercial or financial relationships that could be construed as a potential conflict of interest.

## Publisher’s Note

All claims expressed in this article are solely those of the authors and do not necessarily represent those of their affiliated organizations, or those of the publisher, the editors and the reviewers. Any product that may be evaluated in this article, or claim that may be made by its manufacturer, is not guaranteed or endorsed by the publisher.

## Supplementary Material

The Supplementary Material for this article can be found online at: https://www.frontiersin.org/articles/10.3389/fpsyg.2022.882029/full#supplementary-material
