# Supplementary material for: Effects of Health Belief About COVID-19 on Knowledge Sharing: The Mediating Role of Self-Efficacy
Source: Front Psychol. 2022 Jul 27;13:882029. doi: 10.3389/fpsyg.2022.882029 (PMC9364767; doi:10.3389/fpsyg.2022.882029)
Supplement: Supplementary file 1 [file Table_1.DOCX]

Supplementary Material

| Construct | Measure | Item | Source |
| --- | --- | --- | --- |
| Formal knowledge sharing | FKS1 | My colleagues and I frequently share information using formal communication channels (e.g., routine meetings and project reports) | Peihan and Ruiquan (2021), Shaker et al. (2007) |
|  | FKS2 | My colleagues and I actively share knowledge in formal settings |  |
|  | FKS3 | My colleagues and I often share information about “changes in customer needs” through formal channels |  |
|  | FKS4 | My colleagues and I often share information about “industry trends” through formal channels |  |
| Informal knowledge sharing | IKS1 | My colleagues and I have informal knowledge exchange channels (e.g., discussions over dinner or chat groups on WeChat) | Peihan and Ruiquan (2021), Shaker et al. (2007) |
|  | IKS2 | My colleagues and I frequently share information using informal communication channels |  |
|  | IKS3 | My colleagues and I actively share knowledge in informal settings |  |
|  | IKS4 | My colleagues and I often share information about “industry trends” through informal channels |  |
| Self-efficacy  (When sharing knowledge, I believe that my abilities can ……) | SEF1 | Help my colleagues solve their problems at work | Bock et al. (2005) |
|  | SEF2 | Help my department improve its workflow |  |
|  | SEF3 | Help my department improve efficiency |  |
|  | SEF4 | Help my department achieve performance goals |  |
| Perceived susceptibility | SUS1 | I think I am more likely to contract COVID-19 than others | Wang et al. (2021) |
|  | SUS2 | I think I am very likely to contract COVID-19 in the future |  |
|  | SUS3 | I am worried about contracting COVID-19 at work |  |
|  | SUS4 | My health condition makes me more susceptible to contracting COVID-19 at work |  |
| Perceived severity | SER1 | My career will be at risk if I contract COVID-19 | Wang et al. (2021) |
|  | SER2 | My relationships with family and friends will be affected if I contract COVID-19 |  |
|  | SER3 | My entire life will change if I contract COVID-19 |  |
